# ASO Author Reflections: Neoadjuvant Immunotherapy for Merkel Cell Carcinoma with Clinically Detected Regional Lymph Node Metastasis?

**DOI:** 10.1245/s10434-024-15564-7

**Published:** 2024-05-31

**Authors:** Jenny H. Chang, Kimberly P. Woo, Samer A. Naffouje

**Affiliations:** Department of General Surgery, Cleveland Clinic Foundation, Cleveland, OH USA

## Past

Merkel cell carcinoma (MCC), although rare, is a clinically aggressive cutaneous malignancy. Prognosis and management of MCC are heavily contingent on the extent of nodal involvement. Patients with clinically detected nodal disease (stage IIIB) have historically had low rates of overall survival because of limited systemic treatment options. However, recent findings from clinical trials in other cutaneous malignancies suggest a role for neoadjuvant immunotherapy (NIO).^1,2^ It is thought that dual activation of the immune system within both the tumor tissue and its microenvironment may result in a more robust host immune response prior to the surgical removal of the tumor.^3^ The most recent trends of NIO use in MCC with clinically detected lymph node metastasis and the impact of NIO on overall survival for these patients have yet to be explored.

## Present

Utilizing the National Cancer Database (NCDB) from 2012 to 2019, we found that use of NIO has increased drastically from 0.5 to >50% for patients with Stage III MCC. Nearly all patients who underwent NIO were immunocompetent. When pathologic outcomes were examined in patients who received NIO, the rate of complete response in the primary tumor was 45.2% and partial response was 17.4%, whereas the rate of no response was 37.4%. Of those who also underwent lymph node dissection, 17.9% had negative nodal status on pathology, although the remaining 82.1% had persistent positive nodal status. In total, complete pathological response of both primary tumor and nodal basin (i.e., pCR or ypT0 ypN0) was seen in 7.2%.^4^ After matching between patients who had NIO and did not, those with NIO had a significantly improved overall survival compared with those patients who did not (median OS not reached vs. 35.0 ± 8.0 months, respectively; *p* = 0.025).^4^

## Future

Our study suggests that NIO in MCC with clinically detected regional lymph node metastasis provides improved OS in addition to promising rates of primary complete response, which could change the profile of surgical resection. These findings support ongoing clinical trials that are exploring the use of NIO in MCC.^5^ Importantly, the adverse events of immunotherapy and the role of other predictive markers (such as PD-L1 status, tumor mutational burden, MCPyV status, T cell immunity), which are not included in the NCDB, will be important considerations when discussing the use of NIO for MCC.
